# Supplementary material for: SLIT2/ROBO1 signaling suppresses mTORC1 for organelle control and bacterial killing
Source: Life Sci Alliance. 2023 Jun 13;6(8):e202301964. doi: 10.26508/lsa.202301964 (PMC10264968; doi:10.26508/lsa.202301964)
Supplement: Supplementary file 7 [file LSA-2023-01964_TableS1.docx]

**Supplementary Table 1: Antibodies used in the study**

| **ANTIBODIES** | **SOURCE** | **CATALOG # and RRID:** | **APPLICATION** |
| --- | --- | --- | --- |
| Mouse Anti β-Actin  Clone AC-15 | Sigma-Aldrich, Oakville, ON, Canada | #A5441  RRID: AB_476744 | Western Blotting (WB):  1:2000 1 h |
| Rabbit Anti p-p70S6K (T389) | Cell Signaling Technology, Danvers, MA, USA | #9205  RRID: AB_330944 | WB: 1:1000 overnight |
| Rabbit Anti Total p70S6K | Cell Signaling Technology, Danvers, MA, USA | #9202  RRID:AB_331676 | WB: 1:1000 overnight |
| Rabbit Anti p-ULK1 (S757) | Cell Signaling Technology, Danvers, MA, USA | #14202  RRID: AB_2665508 | WB: 1:1000 overnight |
| Rabbit Anti Total ULK1  Clone D8H5 | Cell Signaling Technology, Danvers, MA, USA | #8054  RRID: AB_11178668 | WB: 1:1000 overnight |
| Rabbit Anti ROBO1 | Thermo Fisher Scientific, Mississauga, ON, Canada | #PA5-29917  RRID: AB_2547391 | WB: 1:500 overnight |
| Rabbit Anti TFEB  Clone D4L2P | Cell Signaling Technology, Danvers, MA, USA | #32361  RRID: AB_2799022 | WB: 1:1000 overnight |
| Rat Anti-Mouse LAMP-1  Clone 1D4B | DSHB, The University of Iowa, Iowa City, IA, USA | #1D4B-s  RRID: AB_2134495 | WB: 1:1000 overnight |
| Rabbit Anti Cathepsin B  Clone D1C7Y | Cell Signaling Technology, Danvers, MA, USA | #31718  RRID: AB_2687580 | WB: 1:1000 overnight |
| Mouse Anti β-tubulin  Clone TUB 2.1 | Sigma-Aldrich, Oakville, ON, Canada | #T4026  RRID: AB_477577 | WB: 1:1000 1 h |
| Rabbit Anti SRGAP2 | Proteintech, Rosemont, IL, USA | # 22519-1-AP  RRID: AB_2879114 | WB: 1:200 overnight |
| Mouse Anti SQSTM1 / p62  Clone 2C11 | Abcam, Toronto, ON, Canada | #ab56416  RRID: AB_945626 | WB: 1:1000 overnight |
| Rabbit Anti LC3B | Novus Biologicals (Bio-Techne Toronto, ON, Canada) | #NB600-1384  RRID: AB_669581 | WB: 1:1000 overnight |
| Rabbit Anti PMP70 | Zymed Laboratories, an Francisco, CA, USA | #71–8300  RRID: AB_87877 | WB: 1:5000 overnight |
| Rabbit Anti PEX14 | Sigma-Aldrich, Oakville, ON, Canada | #ABC142  RRID: AB_2922901 | WB: 1:1000 overnight |
| Mouse Anti GAPDH  Clone 6C5 | Sigma-Aldrich, Oakville, ON, Canada | #MAB374  RRID: AB_2107445 | WB: 1:1000 1 h |
